# Sequential estimation of the generated curing heat of composite materials by data assimilation: A numerical study

**DOI:** 10.1016/j.heliyon.2020.e05147

**Published:** 2020-10-05

**Authors:** Ryota Yokoyama, Ryosuke Matsuzaki, Tadahiro Kobara, Kentaro Takahashi

**Affiliations:** Department of Mechanical Engineering, Tokyo University of Science, Japan

**Keywords:** Materials science, Mechanical engineering, Heat transfer, Materials characterization, Materials mechanics, Solid mechanics, Composite materials, Data assimilation, Process simulation, Process monitoring

## Abstract

The models and parameters related to the generated curing heat in the molding simulation of composite materials are dependent on the type of resin used and the experimental conditions. Therefore, in this study, we estimated the generated curing heat that changes with time by a data assimilation method, which combines the observation values with simulation values, so that the heat curing simulation of carbon fiber reinforced polymers (CFRPs) becomes closer to the experimental conditions. In the data assimilation method, the temperature distribution on the surface of the composite material was used as an observation value, and the generated curing heat was estimated using an ensemble Kalman filter. By optimizing the data assimilation parameters in advance using the response surface method and estimating the generated curing heat by numerical experiments, the generated curing heat could be estimated with an accuracy represented by the time mean error of less than 6%.

## 1 Introduction

The prediction of the molding state of carbon fiber reinforced polymers (CFRPs) by numerical simulation is considered effective for establishing a method to mold high-quality composite materials [1, 2, 3], which in turn requires accurate modeling of the thermal behavior and an understanding of the model parameters. However, given the numerous types of resins and the problem of resin aging, high-precision estimation using only numerical simulations is difficult [4]. Therefore, to understand the molding state of a structure, the temperature and strain, which comprise the heat of the curing reaction during resin molding, were measured by embedding sensors in the structure [5, 6], and the process was monitored. Research has been conducted on smart manufacturing technologies, in which optimal heating control is achieved by process monitoring, which considers the changes in the internal physical properties of the structure [7]. However, monitoring the entire area of a composite material from only the data of the molded surface is difficult. Moreover, there is the concern that the embedded sensors might affect the quality of the molded product.

In contrast to the measurements taken by surface-mounted sensors, which provide only limited information, the data assimilation method [8, 9, 10, 11, 12], which integrates the predicted values obtained by numerical simulation with the observation values obtained from experiments, provides a more accurate estimation of the internal state. Data assimilation methods were originally developed for meteorology [13], oceanography [14], and geology [15] since it enables an estimation of the interested parameters across the full field by integrating discrete observed values and numerical models [15]. Recently, the data assimilation method has been applied to data-based engineering fields [16]. Kato et al. [17] integrated the predicted values obtained by computational fluid dynamics (CFD) simulations with the observation values obtained by wind tunnel experiments by using a data assimilation method to make precise predictions for a complex turbulence model. Hu et al. [18] estimated the inhomogeneous permeability distribution of groundwater using an ensemble Kalman filter (EnKF), which is one of the methods of data assimilation. Wang et al. [19] used non-invasive body surface potential information to estimate the three-dimensional volumetric myocardial membrane potential dynamics. Yardim et al. [20] used a data assimilation technique for source tracking, estimation of environmental parameters of time and space, and frequency tracking in ocean acoustics. Baraldi et al. [21] used data assimilation methods to improve the accuracy of predicting the remaining useful life of a turbine blade affected by creep. Thus, the application of data assimilation methods has expanded to include a variety of fields such as meteorology, fluid mechanics [22], petroleum engineering [23], biomechanics, marine engineering, and materials science.

Data assimilation, which is capable of estimating simulation parameters, has so far been effectively applied to address the problems of estimating the penetration coefficient in the resin impregnation molding of CFRPs [24, 25] and estimating the temperature and thermal conductivity during heat curing [26, 27, 28, 29]. In previous studies [27, 28], the model parameter that is indirectly estimated from temperature observations is thermal conductivity, a value that is constant. Therefore, the effects of the time step and system noise of the variables for data assimilation have not been fully considered. However, the dynamic parameters of the curing heat that is constantly changing in the interior have not been sequentially sequential analyzed in the simulation of CFRP heat curing. In particular, variations in the generated internal heat that depends on the type of resin used and the experimental conditions make it difficult to set up a rational model that reproduces the experiment for simulations, and it is expected that estimation will be possible by data assimilation. However, the system noise and assimilation time steps that are introduced in the time evolution are believed to greatly affect the accuracy of the estimated dynamic state variables, and suitable methods for determining the system noise and assimilation time step have not been built yet.

**Figure 1:**
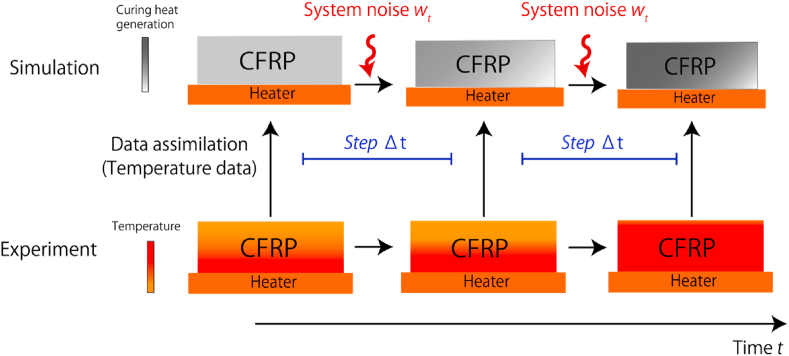
Progressive parameter estimation of CFRP curing using data assimilation.

Therefore, in this study, we incorporated data assimilation into the simulation of CFRP heat curing and evaluated the effect of the data assimilation parameters on the estimation accuracy of the generated curing heat, which varies as the material cures. Furthermore, we propose a method to determine the appropriate data assimilation parameters (Figure 1). In addition to estimating the dynamic state variables, static model parameters such as the heat transfer coefficient between the composite laminate and air and between the composite laminate and the heater, were estimated. These parameters were verified through numerical experiments in which the correct values were already known, and thus the accuracy of the estimation could be verified.

## 2 Simulation of CFRP heat curing

The molding of thermosetting resin composite materials was simulated by modeling the thermal conduction and cure reaction [28]. The relationship between heat flux and temperature in the thermal conduction of anisotropic materials such as CFRPs follows Fourier's law. The non-stationary anisotropic heat transfer function without including the convection term is given by Eq. (1).

(1) $$\rho c\frac{\partial T}{\partial t}=\frac{\partial }{\partial x}\left(k_{xx}\frac{\partial T}{\partial x}\right)+\frac{\partial }{\partial y}\left(k_{yy}\frac{\partial T}{\partial y}\right)+\frac{\partial }{\partial z}\left(k_{zz}\frac{\partial T}{\partial z}\right)+\rho_{r}V_{r}\dot{Q}$$

Here, *T* is the temperature, *t* is the time, *ρ* is the density of the composite material, *ρ*_*r*_ is the density of the resin, *c* is the specific heat capacity of the composite material, and *V*_*r*_ is the volume ratio of the resin in the composite material. Moreover, *k*_*xx*_, *k*_*yy*_, and *k*_*zz*_ denote the thermal conductivity in each of the Cartesian coordinate directions, while $\dot{Q}$ is the curing heat per unit time generated by the curing of thermosetting resins, and is expressed by Eq. (2).

(2) $$\dot{Q}=\frac{dQ}{dt}=H_{r}\frac{d\alpha }{dt}$$

Here, *α* is the degree of curing and *H*_r_ is the total heat of reaction.

The curing reactions of the thermosetting resin are expressed as curing rules using the equations of the Kamal and Sourour model and the Arrhenius equation, as shown in Eqs. (3) and (4) [30].

(3) $$\frac{d\alpha }{dt}=\left(K_{1}+K_{2}\alpha^{m}\right){\left(1-\alpha \right)}^{n}$$

(4) $$K_{i}=A_{i}\text{exp}\left(\frac{-\Delta E_{i}}{RT}\right)\;\left(i=1,2\right)$$

Here, *m* and *n* denote the order of reaction, *K*_*i*_ is the reaction speed constant, *A*_*i*_ is the frequency factor, Δ*E*_*i*_ is the activation energy, and *R* is the gas constant.

Applying numerical discretization of the finite element method [31, 32] and time difference by the implicit solution of the Crank–Nicholson method [33] to solve the differential equation of the non-stationary anisotropic heat conduction equation, the following equation is obtained.

(5) $$\left(\frac{1}{2}K+\frac{1}{\text{Δ}t}C\right)\phi_{t}=\left(-\frac{1}{2}K+\frac{1}{\text{Δ}t}C\right)\phi_{t-1}+\frac{f_{t}+f_{t-1}}{2}\;$$

Here, **K** is the element of the heat transfer matrix, **C** is the element of the thermal capacity matrix, and **f** is the element of the heat flux vector, which are expressed as follows.

(6) $$K=\underset{A}{\int }\left(k_{xx}\frac{\partial \left\{N\right\}}{\partial x}\frac{\partial {\left\{N\right\}}^{T}}{\partial x}+k_{yy}\frac{\partial \left\{N\right\}}{\partial y}\frac{\partial {\left\{N\right\}}^{T}}{\partial y}\right)dA+\;\underset{A_{3}}{\int }h\left\{N\right\}{\left\{N\right\}}^{T}$$

(7) $$C=\underset{A}{\int }\rho c\left\{N\right\}{\left\{N\right\}}^{T}\;dA$$

(8) $$f=\underset{\text{A}}{\int }\rho_{r}V_{r}\dot{Q}{\left\{N\right\}}^{T}dA-\int q_{0}\left\{N\right\}dA+\underset{A2}{\int }\left\{N\right\}dA+\underset{A3}{\int }hT_{fluid}\left\{N\right\}dA$$

Here, ϕ is the nodal temperature, **N** is the interpolation function, and *T*_*fluid*_ is the temperature of the surrounding fluid. The temperature, *T*_1_, is assigned as the Dirichlet boundary condition at boundary *A*_1_. At boundary *A*_2_, the heat flux, *q*_0_, flows in and out according to the Neumann boundary condition. It is further assumed that heat transfer takes place between the solid surface and the surrounding fluid at boundary *A*_3_. It should be noted that the boundary condition used in this study as the heat transfer boundary condition between the parts and surroundings is *A*_3_. From Eq. (5), the heat curing of the thermosetting resin composite material is simulated by considering the generation of internal heat.

## 3 Estimation of CFRP heat curing molding state using EnKF

**Figure 2:**
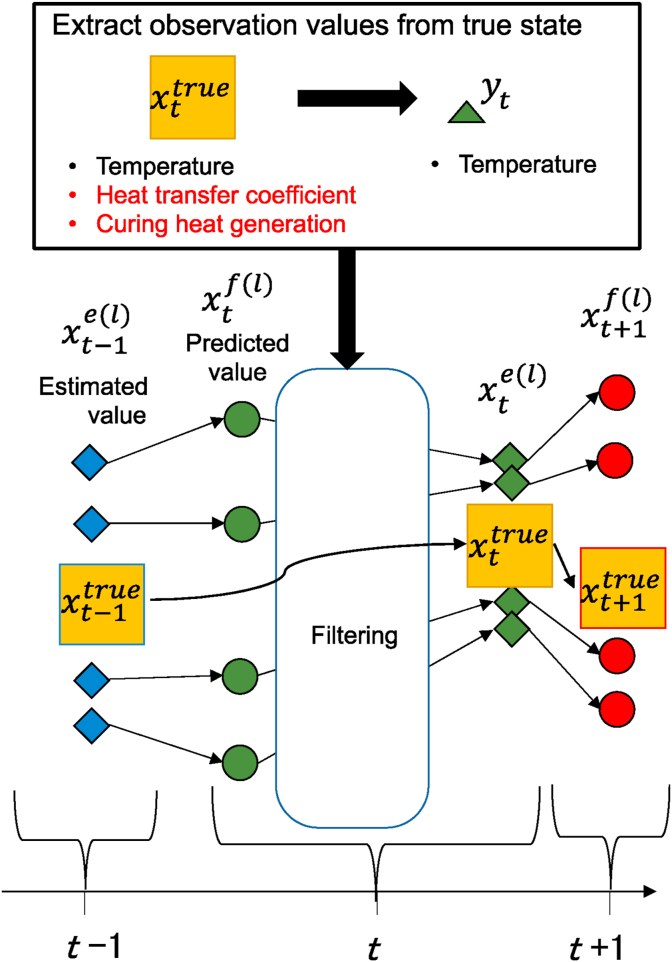
Schematic of the ensemble Kalman filter (EnKF).

Since the heat curing simulation used in this study entails nonlinear time evolution, the EnKF [27], which supports nonlinear models, is believed to be an effective method for state estimation by data assimilation. The EnKF estimates the true state by assimilating the observed values obtained from measurements with the predicted values of the multiple ensembles of time evolution. Figure 2 shows a schematic diagram of the EnKF calculation [28].

The system state transition of the numerical simulation when the state vector at time *t* is **X**_*t*_ for the nonlinear model is expressed by the following system state equation:

(9) $$X_{t}=F_{t}\left(X_{t-1},w_{t}\right)$$

where *F*_*t*_ is the time evolution operator (equivalent to Eq. (5)) and **w**_**t**_ is the system noise. In this study, the system noise is set by the following expression:

(10) $$w_{t}=w_{t}\left(R_{t}\right),$$

where **R**_**t**_ is a vector of the standard normal distribution represented by (0,1^2^).

The state vector **X**_*t*_ is the sum of the nodal temperature vector (**ϕ**_*t*_), heat transfer coefficient (*h*_*air*_ and *h*_*heat*_), and generated curing heat ($\dot{Q}$), as shown in the following expression.

(11) $$\left\{ \begin{array}{c} X_{t}=\left\{\begin{array}{c} \phi_{\text{t}}\\ h_{\mathrm{air}}\\ h_{\mathrm{heat}} \end{array}\right\}\;if\;estimating\;heat\;transfer\\ X_{t}=\left\{\begin{array}{c} \phi_{\text{t}}\\ \dot{Q_{1}}\\ \vdots \\ \dot{Q_{\mathrm{ne}}} \end{array}\right\}\;if\;estimating\;heat\;generation \end{array}\right.$$

Here, *ne* is the number of elements.

The relationship between the state vector (**X**_*t*_) and observed value (**Y**_*t*_) is given by the following observation equation.

(12) $$Y_{t}=HX_{t}+r_{t}=\left\{\begin{array}{c} \left[H0\right]\left\{\begin{array}{c} \phi_{\text{t}}\\ h_{\mathrm{air}}\\ h_{\mathrm{heat}} \end{array}\right\}+r_{t}\;if\;estimating\;heat\;transfer\\ \left[H0\right]\left\{\begin{array}{c} \phi_{\text{t}}\\ \dot{Q_{1}}\\ \vdots \\ \dot{Q_{\mathrm{ne}}} \end{array}\right\}+r_{t}\;if\;estimating\;heat\;generation \end{array}\right\}=\bar{H}X_{t}+r_{t}$$

Here, **H** is the observation matrix that maps the state vector to the observation value, **r**_*t*_ is the observation noise, and the observation value **Y**_*t*_ is expressed as follows.

(13) $$Y_{t}=\left\{\begin{array}{c} T_{t}^{1}\\ T_{t}^{2}\\ \vdots \\ T_{t}^{i}\\ \vdots \\ T_{t}^{N_{o}} \end{array}\right\}$$

Here, *T*_*t*_ is the observation temperature, subscript _*t*_ is the measurement location, and *N*_*o*_ is the number of observations.

Furthermore, using the prediction covariance error matrix ${\bar{P}}_{t}^{\text{f}}$, the observation error covariance matrix **R**, and the Kalman gain ${\bar{G}}_{t}$, we combine the observed value **Y**_*t*_ and predicted value $X_{t}^{f\left(l\right)}$ obtained from Eq. (9) to derive the ensemble estimate $X_{t}^{e\left(l\right)}$.

(14) $${\bar{P}}_{t}^{f}=\frac{1}{L-1}\overset{L}{\underset{l=1}{\sum }}\left(X_{t}^{f\left(l\right)}-{\bar{X}}_{t}^{f}\right){\left(X_{t}^{f\left(l\right)}-{\bar{X}}_{t}^{f}\right)}^{\text{T}}$$

(15) $${\bar{G}}_{t}={\bar{P}}_{t}^{f}H^{\text{T}}{\left(H{\bar{P}}_{t}^{f}H^{\text{T}}+R\right)}^{-1}$$

(16) $$X_{t}^{e\left(l\right)}=X_{t}^{f\left(l\right)}+{\bar{G}}_{t}\left(Y_{t}+r_{t}^{\left(l\right)}-HX_{t}^{f\left(l\right)}\right)$$

Here, the superscript (*l*) denotes the value of the ensemble member.

The average value (${\bar{X}}_{t}^{e}$) is calculated from the ensemble estimated value ($X_{t}^{e\left(l\right)}$) and is used as the estimated value at time *t*.

(17) $${\bar{X}}_{t}^{e}=\frac{1}{L}\overset{L}{\underset{l=1}{\sum }}X_{t}^{e\left(l\right)}$$

where *L* is the number of ensemble members. Basically, the larger the ensemble size, the better the accuracy, but there is a trade-off with computational cost. The optimal number of ensembles was determined for thermal conductivity estimation, and it was discovered that the minimum ensemble size with reasonable accuracy can be determined from the complexity of the analysis target [29].

For a detailed description of the algorithm that estimates the flow state by EnKF, see Reference [34].

Thus, the heat transfer coefficient and the generated curing heat are estimated using the heat-curing simulation and EnKF algorithm.

## 4 Heat transfer coefficient estimation by data assimilation

### 4.1 Observation value analysis model and analysis conditions

**Figure 3:**
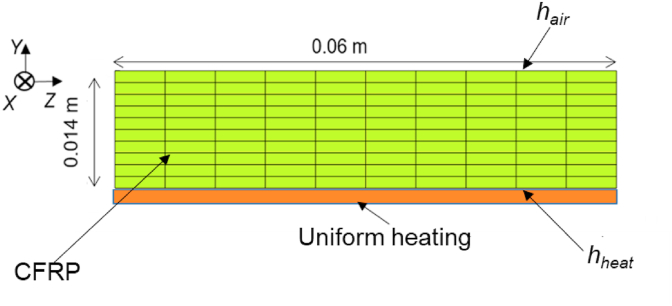
Rectangle model with uniform thermal conductivity.

**Figure 4:**
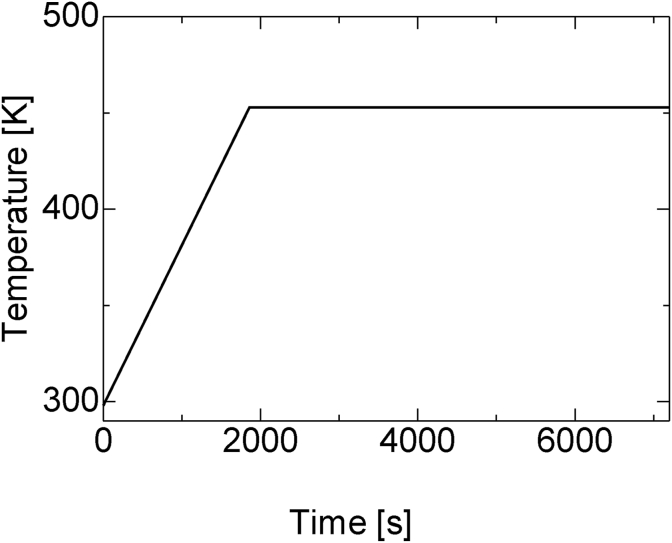
Heating schedule for simulation of CFRP curing.

First, the static parameter of the heat transfer coefficient between the composite material and the external environment was estimated for validation. When verifying by experiment, the true values of the heat transfer coefficient and generated internal heat cannot be obtained. To properly evaluate the accuracy of data assimilation, we did not perform actual experiments to obtain the observed values, but rather used virtual experiments through numerical simulations based on Table 1. The analysis was performed using the rectangle model with uniform thermal conductivity, as shown in Figure 3. The heat transfers between air and the heater at the upper and lower surfaces of the rectangle model were defined as the boundary conditions. The heating schedule consisted of uniformly raising the temperature of the heater installed at the bottom of the composite material to 453 K at a rate of 5 K/min, as shown in Figure 4.

**Table 1:** Cure kinetics parameters of epoxy resin.

Description	Parameter	Value
Total heat of reaction	*H*_r_ [J/kg]	4.63×10^5^
Pre-exponential coefficient	*A*_1_ [1/s]	42.9
	*A*_2_ [1/s]	3.97×10^5^
Activation energy	*ΔE*_1_ [J/mol]	4.37×10^4^
	*ΔE*_2_ [J/mol]	6.99×10^4^
Reaction order	*m*	1.08
	*n*	2.66
Universal gas constant	*R* [J/(mol·K)]	8.3145
Heat transfer coefficient	*h*_air_ [W/m^2^·K]	5.0
Heat transfer coefficient Heating temperature rising	*h*_heat_ [W/m^2^·K]	50
	*T*_heat_ [K/min] (up to 453 K)	5.0
Thermal conductivity of CFRP	*k*_*xx*_ [W/m·K]	0.7
	*k*_*yy*_ [W/m·K]	0.7
Density of CFRP	*ρ* [kg/m^3^]	1540
Specific heat of CFRP	*c* [J/kg·K]	1040
Thermal conductivity of epoxy resin	*k* [W/m·K]	0.2
Density of epoxy resin	*ρ*_*r*_ [kg/m^3^]	1200

The temperatures at 20 points comprising 10 points each on the upper and lower surfaces of the composite laminate were acquired as observation values for the true value model in Figure 3. The heat transfer coefficients defined on the upper and lower surfaces of the laminate, between air and the laminate, and between the heater and the laminate were estimated by EnKF based on Eq. (16). The ensemble member was assigned twice the true values of the heat transfer coefficients, *h*_*air*_ and *h*_*heat*_, and we added 50 normally distributed random numbers with variances of *h*_*air*_ and *h*_*heat*_. The data assimilation parameters are shown in Table 2 and estimation was performed with a time step of 4 s and *w*_*t*_ of 0 W/kg.

**Table 2:** Parameters of the numerical analysis of data assimilation by using EnKF.

Description	Parameter	Value
Analysis time	*t* [s]	7200
Numerical simulation interval	Δ*t* [s]	1.0, 4.0, 8.0, 10.0
Ensemble number	*e*_*n*_	50

The time evolution of the estimation accuracy of the heat transfer coefficient was evaluated using the error rate of Eq. (18).

(18) $$\varepsilon_{t}=\frac{X_{\text{t}}^{\text{true}}-X_{\text{t}}^{\text{a}}}{X_{\text{t}}^{\text{true}}}$$

Further, to evaluate the estimation accuracy between the models, we used the time mean error (TME) of Eq. (19), which is the time average of the interval in which the cure reaction is large.

(19) $$TM\text{E}=\frac{1}{t_{2}-t_{1}}\overset{t_{2}}{\underset{t=t_{1}}{\sum }}\left|\varepsilon_{t}\right|$$

Here, *t*_1_ is 2000 s and *t*_2_ is 4000 s, which are the start and end times of evaluation, respectively.

### 4.2 Results

**Figure 5:**
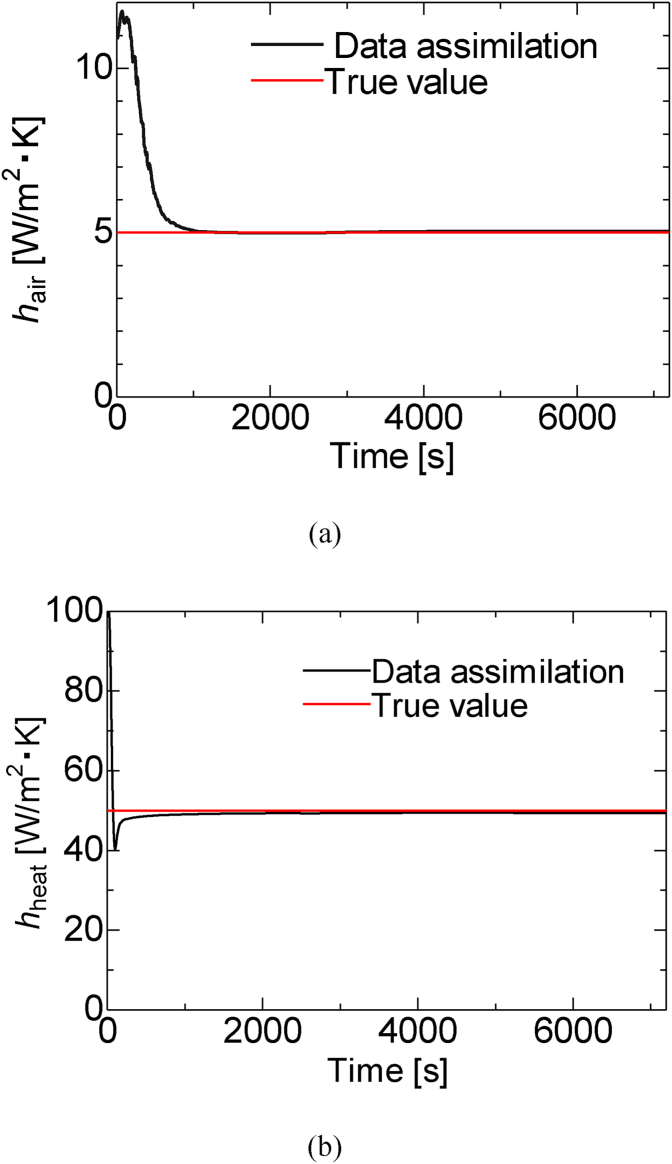
True value and predicted value by data assimilation of the heat transfer coefficients: (a) *h*_air_ and (b) *h*_heat_.

Figure 5 (a, b) shows the results of the estimation of the heat transfer coefficient by data assimilation. As illustrated in Figs. 5a and b, the *h*_*air*_ value was far from the true value at the beginning, but it gradually approached the true value at approximately 1000 s. By contrast, *h*_*heat*_ rapidly approached the true value, reaching it at 1000 s, and then overshooting it. Nevertheless, the estimated values are closer to the true value by 1000 s when the generated curing heat starts to increase. The longer assimilation time of *h*_*air*_ is possibly because the temperature takes a longer time to transmit to the surface.

**Figure 6:**
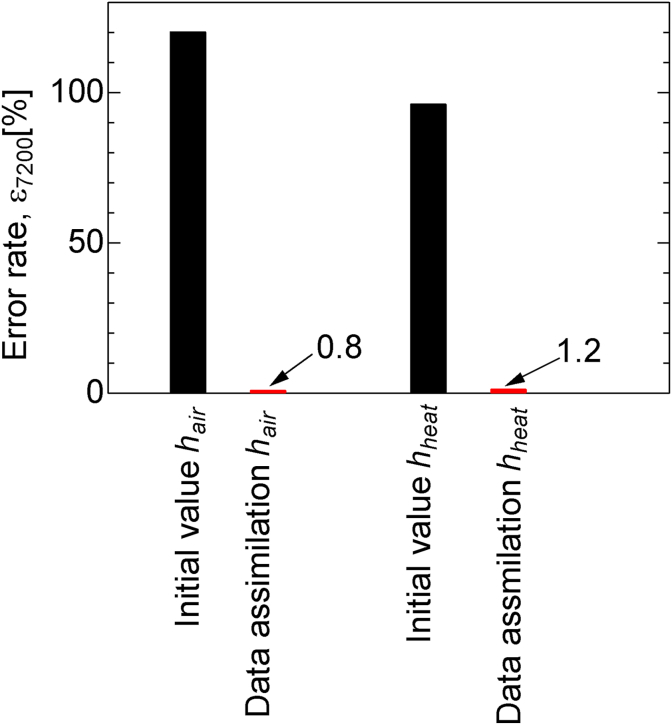
Error rate of the heat transfer estimation for the initial value and the data assimilated value at 7200 s.

Figure 6 shows the error rates of the initial value and estimation value at 7200 s for the ensemble member. As illustrated in Figure 6, the initial error rate was 120 % for *h*_*air*_ and 96 % for *h*_*heat*_ because we used twice the true value of the heat transfer coefficient as the average of the initial values of the heat transfer coefficient of the ensemble members. The error rate of the true value at 7200 s was 0.8% for *h*_*air*_ and 1.2% for *h*_*heat*_; this indicates that the estimation errors reduced by 119.2% and 95.8%, respectively.

Thus, the heat transfer coefficient, which is the boundary condition, can be estimated even when the heat transfer coefficient estimation by data assimilation is assigned an initial value that is twice the true value.

## 5 Estimation of the generated curing heat by data assimilation

### 5.1 Impact assessment of data assimilation parameters

Next, we carried out data assimilation estimation of the generated curing heat, which is a dynamic parameter that varies depending on the degree of curing and affects the temperature of the laminate. The true value model that was used is the same as that described in Section 4.

According to the system state equation for data assimilation, the amount of generated internal heat ($\dot{Q}$) of Eq. (1) was estimated by data assimilation without calculating Eqs. (2), (3), and (4). In this analysis, the parameters in Table 2 were used to set the time steps to 1 s and 4 s, and the system noise *w*_*t*_ to 0 W/kg and 2 W/kg.

**Figure 7:**
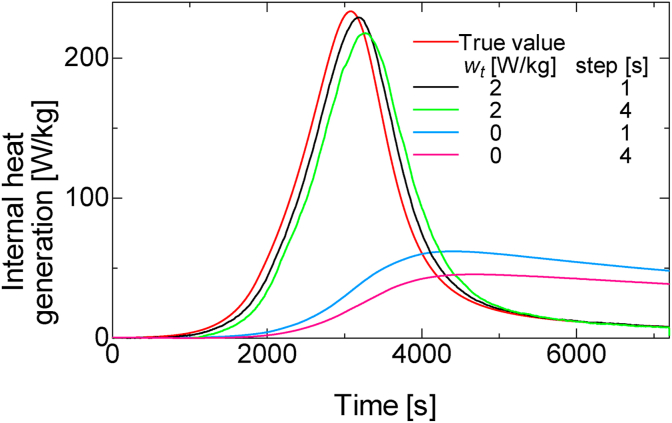
Data assimilation results and true value of the internal heat generated during curing.

**Figure 8:**
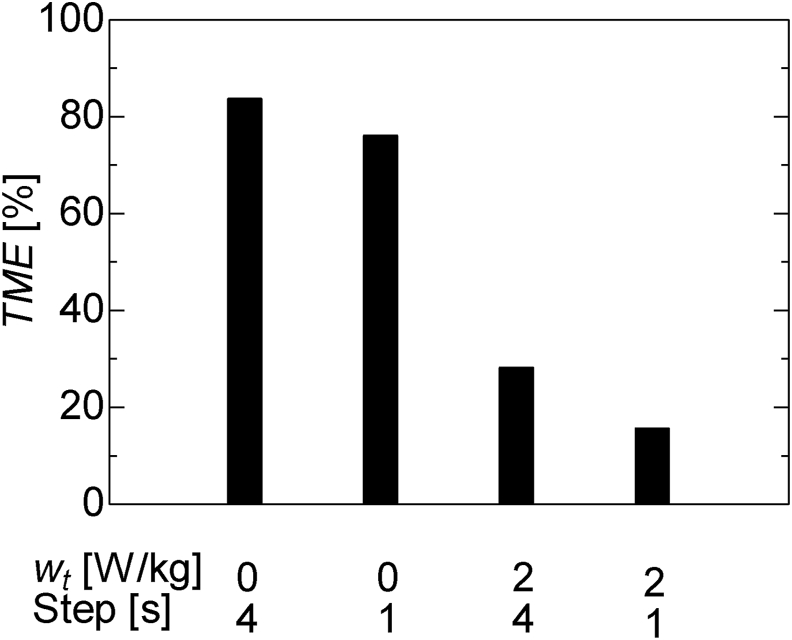
Time mean error of data assimilation result with various data-assimilation parameters during 2000–4000 s.

Figure 7 shows the data assimilation analysis results. The comparison of the time steps 1 s and 4 s with the same system noise shows that at a time step of 1 s where the number of data assimilation increases, the estimated value is closer to the true value, which indicates improved estimation accuracy. Further, a comparison of the graphs of *w*_*t*_ of 0 W/kg and 2 W/kg showed that the estimate that qualitatively defines the system noise is closer to the true value. Figure 8 shows the TME with the true value of various parameters from 2000 s to 4000 s, where the change in the true value of curing heat is large. These results show that the quantitative accuracy of estimation increases when the time step is 1 s and *w*_*t*_ is 2 W/kg.

This discussion demonstrates that it is possible to estimate the internal heat generated by heat curing using data assimilation. In addition, the system noise and time step were found to affect the estimation accuracy of the dynamic parameters; hence, an appropriate setting method is necessary. When the time step of data assimilation is large, it is assumed that the generated internal heat that changes with time in practice, is constant, and there is a delay in the estimation of the actual phenomenon. Therefore, a slight time step setting is necessary to estimate the parameters that are dynamic. Meanwhile, if the time step is very short, the numerical simulation and data assimilation process of the ensemble member may take longer than the actual time of the phenomenon. Therefore, it is important to model a minimum time step that is within the time range to observe the actual phenomenon.

### 5.2 Determination of data assimilation parameters by response surface method

The response surface method [34] is used to determine the system noise and time step appropriately. A response surface is an approximation of the function of the response *y* predicted from *n* predictor variables *x*_*i*_ (*i* = 1,…,*n*) (*n* > 1).

(20) $$y=f\left(\;x_{1},\ldots ,x_{n}\right)+\varepsilon$$

Here, *ε* is the error.

In the response surface method, an approximation function is used for optimization regardless of the shape of the function. This may be a linear function or a function that can be linearized and easily estimated statistically using the least squares method. Moreover, the approximation function can be statistically evaluated. The function of the response surface used in this study is a second-order polynomial, with the system noise and time step as design variables and the response as estimation accuracy, and is expressed as follows.

(21) $$y=\beta_{0}+\beta_{1}x_{x}+\beta_{2}x_{2}+\beta_{3}x_{1}^{2}+\beta_{4}x_{2}^{2}+\beta_{5}x_{1}x_{2}$$

Since the error sum of the squares is minimized, the unbiased estimator (*b*) of coefficient *β* is obtained using the following equation [34].

(22) $$b={\left(X^{\text{T}}X\right)}^{-1}X^{\text{T}}y,$$

where **X** and **y** are the data matrix of the design variables and the data vector of the response, respectively. The superiority of each coefficient in the regression model was determined by the regression coefficient t-test. The hypothesis that the *j*-th coefficient *β*_*j*_ = 0 of the regression equation, that is, the *j*-th variable does not contribute to the regression variable is tested. In this study, factors with a p-value of 0.5 or more are eliminated and reanalysis is performed. This process is repeated until the p-value becomes less than 0.5. Data assimilation parameters are determined by finding design variables that minimize the response of the obtained response surface.

In this study, optimization was performed to improve the accuracy of heat generation, which has been greatly reduced by the time step and system noise settings. The heat transfer coefficient can also be added to the objective function; however, because it becomes multi-objective functions, the optimal solution becomes a Pareto solution and the optimal variable cannot be uniquely determined. In this case, we treated heat generation as the only objective function, as data assimilation variables have a strong influence on heat generation, which is dynamic.

### 5.3 Determination and evaluation of data assimilation parameters

Since the time step and system noise discussed in Section 5 affect the estimation accuracy of the dynamic parameters of data assimilation, the time step was set to 1, 4, 8, and 10 s, and *w*_*t*_ was set to 2, 4, 10, 20, 30, 40, 50, 60, 70, 80, 90, and 100 W/kg, and three rounds of analyses were conducted for each of the set values. The average of the three analyses was used to determine the TME [%] in the interval of 2000 s–4000 s. A second-order polynomial response surface, with the data assimilation estimation accuracy as the response and the time step and system noise as the design variables, was obtained.

(23) $$TME=10.56-0.1393\times wt+1.160\times step+{\left(wt-47.44\right)}^{2}\times 0.003478+\left(wt-47.44\right)\times \left(\left(step-5.75\right)\times \left(-0.01332\right)\right)+{\left(step-5.75\right)}^{2}\times \left(-0.03810\right)$$

**Figure 9:**
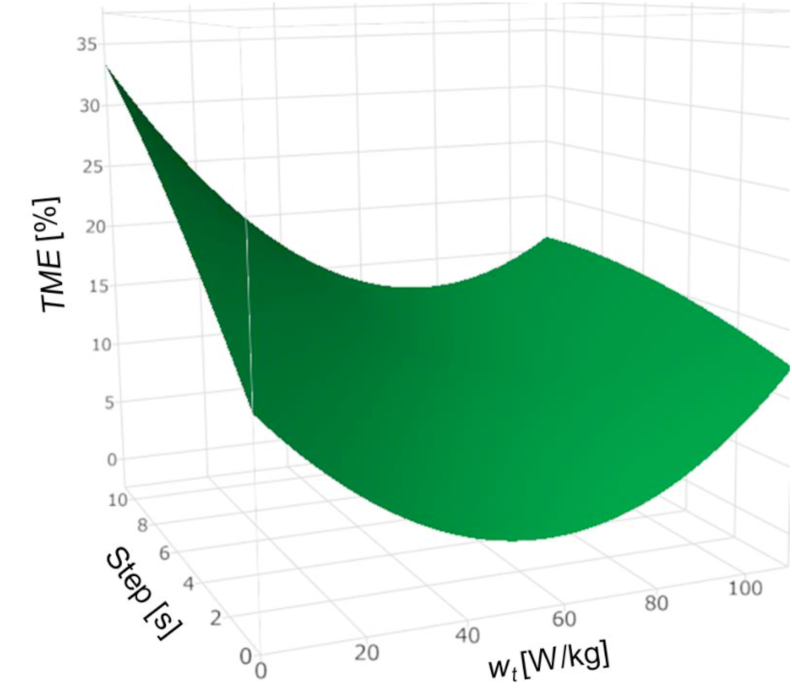
Constructed response surface using various steps and system noise *w*_*t*_.

Figure 9 shows the resulting response surface. The adjusted R^2^ value of the degrees of freedom of the response surface was 0.87, indicating that it was well approximated by a second-order polynomial. The minimization of Eq. (23) under the constraint of step ≥1 yielded the values *w*_*t*_ = 58.37 [W/kg] and step = 1 [s]. Because the optimization uses quadratic polynomial response surface methods, its minimum value corresponds to the vertex of the quadratic polynomial, and the solution is obtained instantaneously compared to commonly-used genetic algorithms.

**Figure 10:**
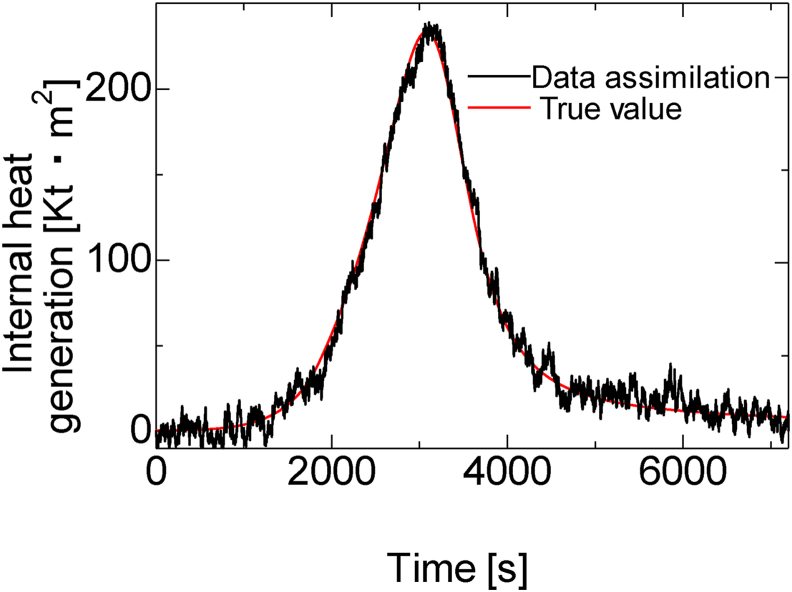
Data assimilation result of the generation of internal heat at optimal conditions (*step* = 1 s, *w*_t_ = 57.54 W/kg).

**Figure 11:**
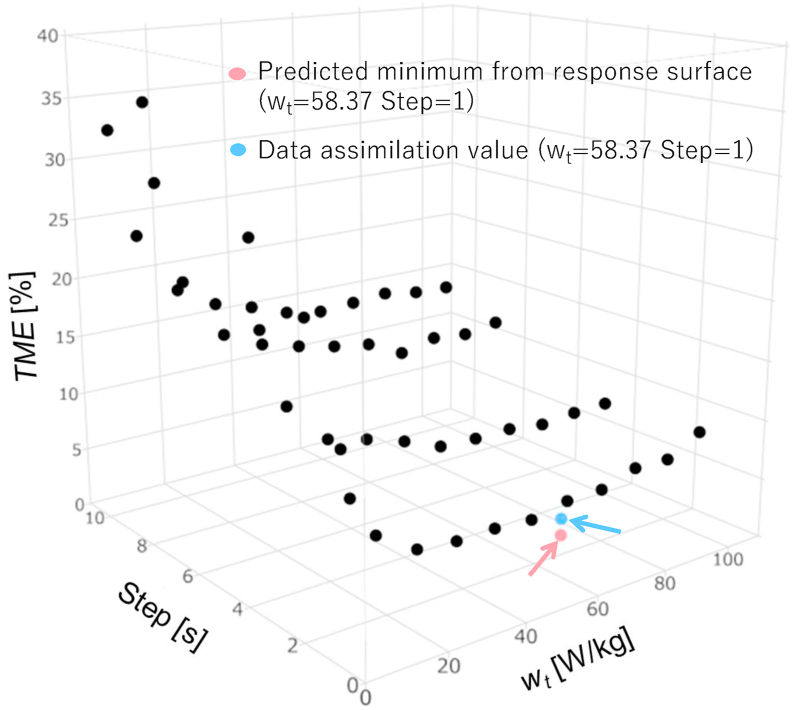
Time mean error of response surface and data-assimilation results with optimal conditions.

Figure 10 shows the results of re-estimation by data assimilation using the data assimilation parameters thus obtained, and Table 3 shows the TME. Although the predicted value and the result of the adjustment are somewhat different, the TME in the interval of 2000 s–4000 s, where the change was large, was estimated within 6%. Thus, the accuracy improved when the time step was closer to 1 s and *w*_*t*_ was found to have an optimal value. The scatter diagram of the analysis results used for this prediction is shown in Figure 11 along with the minimum predicted values and the actually applied results. These results indicate that the actually applied results were more accurate than the TME of all the experimental points used to create the response surface. This demonstrates the effectiveness of the minimization search using the prediction by the surface response method.

**Table 3:** Forecast result and data assimilation value.

	*w*_*t*_ = 58.37 [W/kg] and *step =*1 [s]
Expected minimum *TME*	3.836
Data assimilation *TME*	5.183

## 6 Conclusion

In this study, estimations were made using two parameters: one is a constant value derived from the heat transfer rate, which is also the boundary condition, and the other is derived by data assimilation estimation based on the generated curing heat, which changes as the material cures. We verified that the heat transfer coefficient could be identified with a TME of 1.2%. In addition, we found that the time step and system noise affect the accuracy of the estimation of the generated curing heat, a parameter that changes with curing, by data assimilation. The finer the time step, the better the accuracy, but one should be careful with the increase in computational time when performing data assimilation in real time. With the elimination of system noise, the variation of the initial ensemble members alone was found to deviate significantly from the actual change in the generated internal heat. These factors were appropriately set using the response surface method and it was possible to obtain estimations with a TME of <6%. This enabled the correct estimation of the heat value that varies with curing by data assimilation, which is expected to lead to a highly accurate prediction of residual strain and deformation due to curing. In this study, the heat transfer coefficient and the generated heat were estimated separately. For further research, we will verify the simultaneous estimation of several parameters including the heat transfer coefficients and the generated heat.

## Declarations

### Author contribution statement

Ryota Yokoyama: Conceived and designed the experiments; Performed the experiments; Analyzed and interpreted the data; Wrote the paper.

Ryosuke Matsuzaki: Conceived and designed the experiments; Analyzed and interpreted the data; Wrote the paper.

Tadahiro Kobara & Kentaro Takahashi: Analyzed and interpreted the data.

### Funding statement

This work was supported by the Cross-ministerial Strategic Innovation Promotion Program (SIP) through the ‘‘Innovative Structural Material Project’’ (Funding agency: JST, Japan).

### Competing interest statement

The authors declare no conflict of interest.

### Additional information

No additional information is available for this paper.
